# Clarifying "never events and introducing "always events"

**DOI:** 10.1186/1754-9493-3-26

**Published:** 2009-12-31

**Authors:** Alan Lembitz, Ted J Clarke

**Affiliations:** 1 Colorado Physician Insurance Company (COPIC), Headquarters, Denver, CO 80230, USA

## 

Despite the widespread usage of the term "never events," the *National Quality Forum *(NQF) refers to these events as "serious reportable events" in all of their definitions and references. In this editorial, we use the popular - but likely improper - term "never events" as it further illustrates the public's perception of adverse occurrences. Although the preferred terminology reverts to "serious reportable events", this definition may be unlikely be given the prevalence of the viscerally moving term "never event."

Further confusion persists about the definition of "never events" as they relate to either (1) conditions listed as "serious reportable events" by the NQF, in contrast to (2) conditions defined by the *Centers for Medicare and Medicaid Services *(CMS) have deemed as "non-reimbursable serious hospital-acquired conditions".

## National Quality Forum (NQF) - definition of "never events"

The NQF is a nonprofit organization that aims to improve the quality of healthcare in the United States http://www.qualityforum.org. In 2002, the NQF published a first report which defined 27 so-called "serious reportable events" in healthcare. These encompass serious adverse events occurring in hospitals that are largely preventable and of concern to both the public and to healthcare providers. One additional event was added to the updated report in 2006, leading to a total 28 "never events" defined by the NQF (table 1) [1,2]. While most on the list of "serious reportable events" include obvious unacceptable errors, such as wrong site surgery or discharge of an infant to the wrong person, not all NQF events are preventable at all times or indicative of obvious negligence [3]. A goal of quality improvement measures should be to institute a reduction of "never events" to zero. Achieving that goal via the cycle of reporting, intervention, and measurement of subsequent outcomes must necessarily begin with a culture of openly reporting these defined events within an institution [4-6].

**Table 1:** Serious reportable events ("never-events"), as defined by the National Quality Forum (NQF consensus report, update 2006; http://www.qualityforum.org)

1.	Surgery performed on the wrong body part.
2.	Surgery performed on the wrong patient.

3.	Wrong surgical procedure performed on a patient.

4.	Unintended retention of a foreign object in a patient after surgery or other procedure.

5.	Intraoperative or immediate postoperative death in an ASA class I patient.

6.	Patient death or serious disability associated with the use of contaminated drugs, devices, or biologics provided by the healthcare facility.

7.	Patient death or serious disability associated with the use or function of a device in patient care in which the device is used or functions other than as intended.

8.	Patient death or serious disability associated with intravascular air embolism that occurs while being cared for in a healthcare facility.

9.	Infant discharged to the wrong person.

10.	Patient death or serious disability associated with patient elopement (disappearance)

11.	Patient suicide, or attempted suicide, resulting in serious disability while being cared for in a healthcare facility.

12.	Patient death or serious disability associated with a medication error.

13.	Patient death or serious disability associated with a haemolytic reaction due to the administration of ABO/HLA-incompatible blood or blood products.

14.	Maternal death or serious disability associated with labor or delivery in a low-risk pregnancy while being cared for in a healthcare facility.

15.	Patient death or serious disability associated with hypoglycaemia, the onset of which occurs while the patient is being cared for in a healthcare facility.

16.	Death or serious disability (kernicterus) associated with failure to identify and treat hyperbilirubinemia in neonates.

17.	Stage 3 or 4 pressure ulcers acquired after admission to a healthcare facility.

18.	Patient death or serious disability due to spinal manipulative therapy.

19.	Artificial insemination with wrong donor sperm or wrong egg.

20.	Patient death or serious disability associated with an electric shock while being cared for in a healthcare facility.

21.	Any incident in which a line designated for oxygen or other gas to be delivered to a patient contains the wrong gas or is contaminated with toxic substances.

22.	Patient death or serious disability associated with a burn incurred from any source while being cared for in a healthcare facility.

23.	Patient death or serious disability associated with a fall while being cared for in a healthcare facility.

24.	Patient death or serious disability associated with the use of restraints or bedrails while being cared for in a healthcare facility.

25.	Any instance of care ordered by or provided by someone impersonating a physician, nurse, pharmacist, or other licensed healthcare provider.

26.	Abduction of a patient of any age.

27.	Sexual assault on a patient within or on the grounds of a healthcare facility.

28.	Death or significant injury of a patient or staff member resulting from a physical assault (i.e. battery) that occurs within or on the grounds of a healthcare facility.

## Centers for Medicare and Medicaid Services (CMS) - definition of "never events"

CMS adopted the non-reimbursement policy for certain "never events" - defined as "non-reimbursable serious hospital-acquired conditions" - in order to motivate hospitals to accelerate improvement of patient safety by implementation of standardized protocols. These newly defined "never events" limit the ability of the hospitals to bill Medicare for adverse events and complications. The non-reimbursable conditions apply only to those events deemed "reasonably preventable" through the use of evidence-based guidelines.

Arnold Milstein, MD, a member of the Medicare Payment Advisory Commission, elaborated on CMS' rationale. Milstein states, *"The new payment approach is actually a relatively small step in a cautious, intermittent, 50-year effort by payers to stimulate U.S. hospitals and clinicians to accelerate improvement in the quality of care and reductions of wasted spending" *[7]. Dr. Milstein goes on to quote Kenneth Kizer, the man who coined the term "never events" while leading the National Quality Forum. Kizer asserts that using the negative carries an extra psychological charge. Dr. Milstein also points to Kahneman and Tversky's Nobel prize winning research on "negative framing" which suggests that humans are more strongly inclined to take action when the actions in question are labeled so as to convey the loss avoided (rather than the benefit gained) and when the consequences of failing to act are mentally vivid [7].

### Liability concerns and negligence claims

The biggest concern we face is the public confusion between the two lists, based on two distinct definitions by the NQF and CMS, respectively. Most, but not all, of the events on the NQF "never events" likely carry liability. While the amount of compensation may be questionable, few argue against the just compensation for injuries that result from never events. However, many of the non-reimbursable CMS "never events" are not completely preventable, even with the best practice of evidence-based treatment. We are concerned that patients experiencing complications listed as "non-reimbursable serious hospital-acquired conditions" will be inaccurately told that those "never events" are based on negligence or medical errors. It won't be long before we see trial lawyers advertise and openly solicit patients with conditions deemed "never events" or "non-reimbursable events".

**Figure 1:**
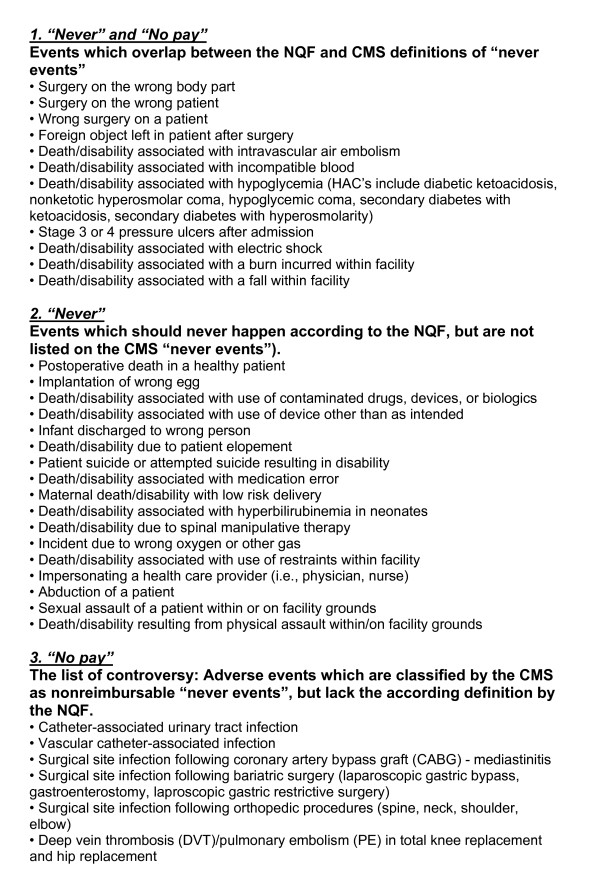
**Comparison of "never events", as defined by the NQF ("serious reportable events") versus CMS ("non-reimbursable serious hospital-acquired conditions")**.

Although many of the events listed by NQF and CMS are preventable, this is not always the case (Figure 1). We wish to emphasize and discuss some examples related to the controversy related to the preventability of some of the listed conditions:

#### (1) Prevention of falls

A recent editorial in the *New England Journal of Medicine *argues that the inclusion of "falls" on both lists is misguided[8]. According to Dr. Sharon Inouye (Harvard Medical School), there is currently no evidence that hospital falls *"(...) can be consistently and effectively prevented through the application of evidence-based guidelines. (...) Their inclusion may have unintended consequences that may cause greater harm than the falls that the initiative is meant to prevent" *[8].

Unintended consequences are likely to include a decrease in mobility, increase in use of physical restraints, and a tendency to focus on measures including new prevention devices. These measures can cause reallocation of resources from areas that might have greater impacts on patient safety. According to the editorial, *"Falls are often the result not of medical errors but of disease, impairments, and appropriate uses of medications and other treatments. Falls and injuries can occur even when hospitals provide the best possible care" *[8].

#### (2) Postoperative infections and thromboembolic events

It is known that certain orthopaedic procedures can result in the hospital-acquired conditions of postoperative infections and thromboembolic events. Neither complication can ever be completely prevented. Arguments also exist that vigorous thromboprophylaxis in certain orthopaedic procedures can lead to an increased risk of delayed bleeding, wound healing problems, and postoperative infections. This notion has led the *American Academy of Orthopaedic Surgeons *(AAOS) to recommend different prophylaxis regimens compared to the evidence-based guidelines published by the *American College of Chest Physicians *(ACCP)[9].

We strongly feel that there is a need to officially clarify that falls, postoperative infections, and thromboembolic events are "non-reimbursable serious hospital-acquired conditions", but not "never events". Efforts certainly must be made to reduce either complication as much as possible via evidence-based assessment and treatment. Documentation of the informed consent process, and the risk/benefit analysis underlying the clinical decision making processes are critical to patient understanding of potential complications and our ability to defend the care provided in the medicolegal setting.

### Strategies to reduce risk

Strategies to improve the defensibility of care where appropriate, particularly those falling under the non-preventable adverse events list include:

• Pretreatment or pre-hospital documentation of underlying pre-existing conditions, particularly those involving infections, pressure sores, altered mental status, hyper-/hypoglycemia, and patients at high risk for venous thromboembolism.

• Hospital outcomes data with identification of care improvements directed at those complications - particularly hospital-acquired infections.

• Standardized and universally followed approaches to reduce wrong site/wrong patient surgery.

• Culture-changing training around communication, assertiveness, team training, and the use of briefings and debriefings, particularly in high-acuity patient care areas.

• The use of surgical checklists.

• Understanding and using clear language in policies and publications of the difference between the NQF "never events" and the CMS "non-reimbursable serious hospital-acquired conditions" to avoid claims of negligence.

### Introducing a positive approach towards patient safety: the "always events"

"Never events" and non-reimbursable adverse events are framed in the negative and likely carry some "extra psychological charge", as mentioned above. Our concept of the "always events" represents a positive affirming behavior that can motivate us to improve patient safety and promote better outcomes. Some basic examples of "always events" include:

• Including patient identification by more than one source.

• Mandatory "readbacks" of verbal orders for high-alert medications.

• Disclosure of adverse outcomes and transparency with patients and families.

• Medication error reduction strategies.

• Surgical time-out.

• Anesthesia monitoring that is appropriate for the level of sedation.

• Tracking of critical imaging, lab and pathology results.

• Making critical information available at handoffs or transitions in care.

Standardization and validation of "always events" may represent the basis for a positive long-term culture of patient safety to be passed on to the next generation of health care providers.

## Competing interests

Both authors are affiliated with the *Colorado Physician Insurance Company *(COPIC). The author declares no other competing interests with regard to this manuscript.

## Authors' contributions

Both authors contributed equally in the design and writing of this editorial.
